# Supplementary material for: The 20-hydroxyecdysone agonist, halofenozide, promotes anti-Plasmodium immunity in Anopheles gambiae via the ecdysone receptor
Source: Sci Rep. 2020 Dec 3;10:21084. doi: 10.1038/s41598-020-78280-8 (PMC7713430; doi:10.1038/s41598-020-78280-8)
Supplement: Supplementary file 1 — Supplementary Information. [file 41598_2020_78280_MOESM1_ESM.pdf]

Supplemental Data

The 20-hydroxyecdysone agonist, halofenozide, promotes anti-*Plasmodium* immunity in *Anopheles gambiae* via the ecdysone receptor

Rebekah A. Reynolds[1], Hyeogsun Kwon[1], Thiago Luiz Alves e Silva[2], Janet Olivas[2], Joel Vega-Rodriguez[2], and Ryan C. Smith[1]*

[1]Department of Entomology, Iowa State University, Ames, Iowa, USA

[2]Laboratory of Malaria and Vector Research, National Institute of Allergy and Infectious Diseases, National Institutes of Health, Rockville, Maryland, USA.

*Correspondence: smithr@iastate.edu

Supplemental Information

Supplemental Figure Legends

Figure S1. Mosquito survival following halofenozide application.

Figure S2. Halofenozide does not influence bacteria.

Figure S3. Increasing concentrations of halofenozide stimulate higher levels of ecdysone signaling.

Figure S4. Targeting the heterodimeric ecdysone receptor (EcR/USP) by RNAi.

Supplemental Tables

Table S1. Primers for qRT-PCR analysis.

Table S2. Primers for dsRNA synthesis.=

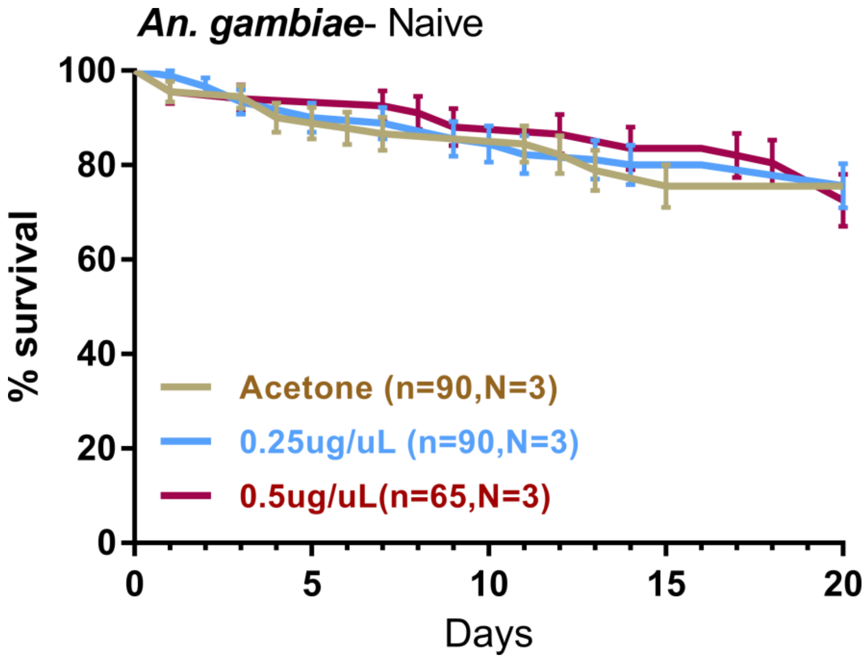

Figure S1. Mosquito survival following halofenozide application. Naive female *An. gambiae* mosquitoes were topically applied with either acetone (control) or halofenozide (0.25 µg/µL and 0.5 µg/µL). Mosquito survival was monitored for 20 days post-application. No significant differences between treatments were detected when a Log-rank (Mantel-Cox) test was performed.

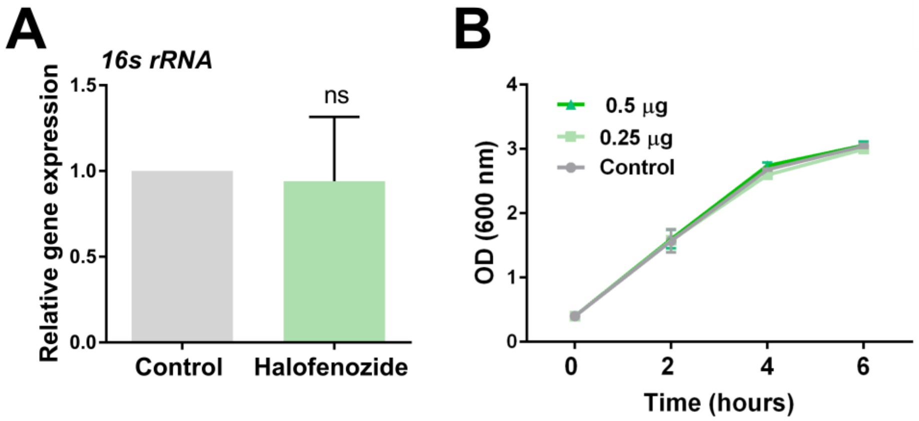

Figure S2. Halofenozide does not influence bacteria. The influence of halofenozide on the mosquito microbiota was examined in control- or halofenozide-treated (0.5 µg) mosquitoes using 16s rRNA primers by qRT-PCR (**A**). Results display previously results from four independent experiments. Data were examined using Mann-Whitney analysis. ns, not significant. Bacteria growth was also examined *in vitro*, in which halofenozide (0.25 and 0.5 µg/ml) or acetone (control) were added to liquid cultures of *E. coli* and their growth was examined for 6 hours using measurements of optical density (OD) at 600 nm (**B**). Experiments were performed in two independent experiments and analyzed using a two-way ANOVA and Bonferroni's multiple comparison test. No significant differences were detected between treatments at any experimental timepoint.

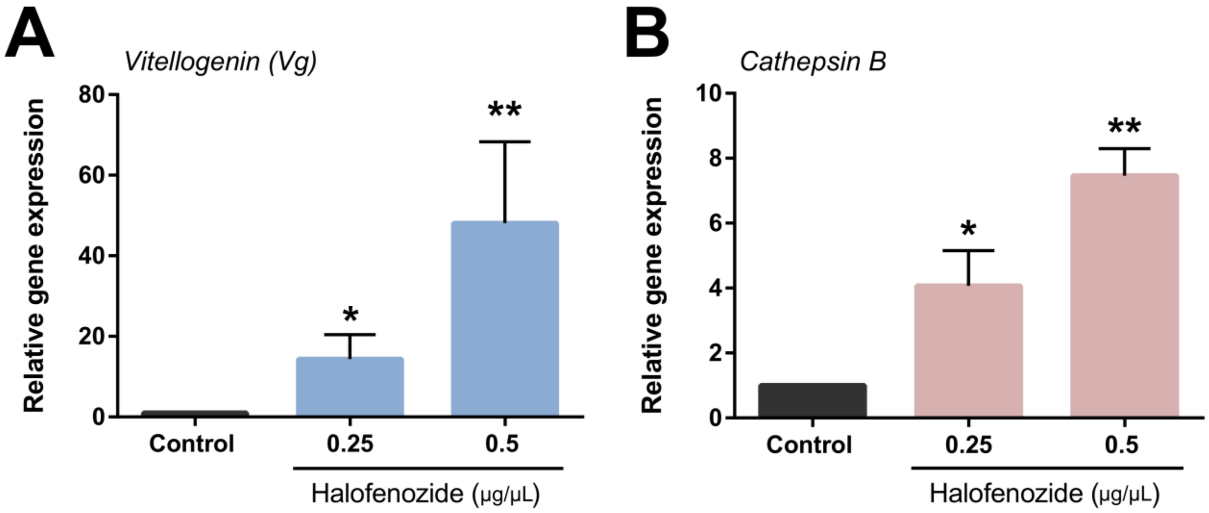

Figure S3. Increasing concentrations of halofenozide stimulate higher levels of ecdysone signaling. Analysis of vitellogenin (A) and cathepsin B (B) gene expression by qRT-PCR in response to increasing concentrations of halofenozide in whole naive female *An. gambiae*. Data from three or more experiments were compared to control (acetone) and analyzed using a Mann-Whitney test in GraphPad Prism 6.0 to determine significance (*, $P<0.05$; **, $P<0.01$).

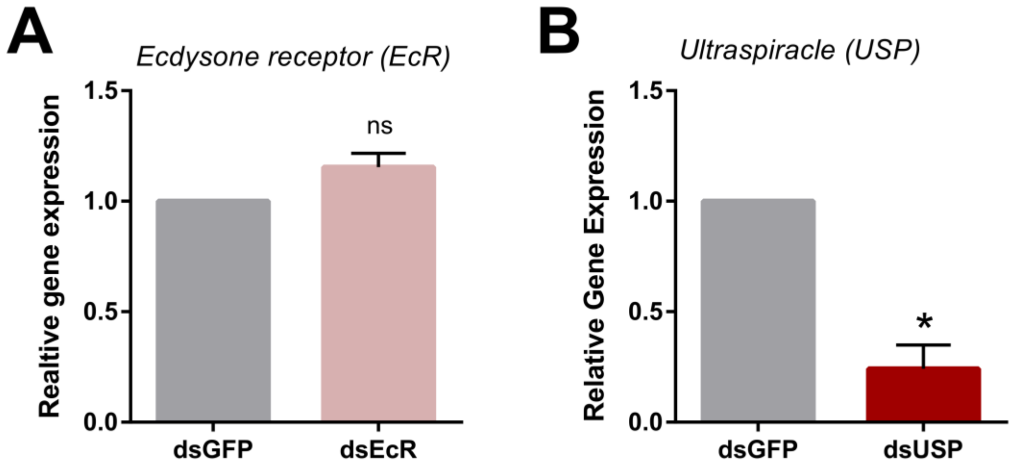

Figure S4. Targeting the heterodimeric ecdysone receptor (EcR/USP) by RNAi. To disrupt the heterodimeric ecdysone receptor (EcR/USP), EcR and USP were individually targeted by the injection of specific dsRNA. To validate the presence or absence of a knockdown, the expression of *EcR* **(A)** and *USP* **(B)** in whole mosquitoes 2 days post-dsRNA injection by qRT-PCR. Data from three or more experiments were analyzed using a Mann-Whitney test in GraphPad Prism 6.0 to determine significance (*, $P<0.05$).

Table S1. Primers for qRT-PCR analysis

Primer	Gene ID	Sequence (5'- 3')
Cathepsin B-F	AGAP004534	GCCAACGGTCTAGTGTCGAGTGTCG
Cathepsin B-R		ACTCGTACCGTCTGATCGGCACCTT
Cecropin 1-F	AGAP000693	TTCATCTTTGTCGTGCTGGC
Cecropin 1-R		GCACTGCCAGCACGACAAAG
Cecropin 3-F	AGAP000694	ACGTACTGAACCACCTGCGCGTT
Cecropin 3-R		GCGCTGTGTGCGCCGATGAA
rpS7-F	AGAP010592	ACCCCATCGAACACAAAGTTGACACT
rpS7-R		CTCCGATCTTTCACATTCCAGTAGCAC
USP-F	AGAP002095	TGAAGTCCGAAGAAATCAACTCGAC
USP-R		GGGCAAACTCGATTAGCTGGTAGAT
Vg-F	AGAP004203	TGCAGTACATCGAGCAGGGTGACAA
Vg-R		CTTGACGGTCTTGGTGACCGACTTG
Universal bacteria 16S-F	N/A	TCCTACGGGAGGCAGCAGT
Universal bacteria 16S-R		GGACTACCAGGGTATCTAATCCTGTT

Table S2. Primers for dsRNA synthesis

Primer	Gene ID	Sequence (5’- 3’)
GFP T7-F		TAATACGACTCACTATAGGGAGAATGGTGAGCAAGGGCGAGGAGCTGT
GFP T7-R		TAATACGACTCACTATAGGGAGATTACTTGTACAGCTCGTCCATGCC
USP T7-F	AGAP002095	TAATACGACTCACTATAGGGCCTAAAATGTCG
USP T7-R		TAATACGACTCACTATAGGGATCGGGAGACACACGCAGT
